# Disseminated *Cryptococcus neoformans* infection involving multiple bones and lung in an immunocompetent patient: a case report

**DOI:** 10.1186/s12879-024-09264-6

**Published:** 2024-04-12

**Authors:** Yang-Ming Lee, Yuag-Meng Liu, Tsung-Chia Chen

**Affiliations:** 1 Department of Internal Medicine, Changhua Christian Hospital, 135 Nanhsiao Street, 500 Changhua, Taiwan; 2 Division of Infectious Diseases, Department of Internal Medicine, Changhua Christian Hospital, Changhua, Taiwan; 3 Division of Infectious Diseases, Department of Internal Medicine, Taichung Hospital, Ministry of Health and Welfare, Taichung City, Taiwan

**Keywords:** *Cryptococcus neoformans*, Cryptococcal osteomyelitis, Immunocompetent, Dissemination

## Abstract

**Background:**

Cryptococcal osteomyelitis is a rare and potentially serious condition, typically encountered in individuals with compromised immune systems. This case underscores the unusual occurrence of disseminated Cryptococcosis in an immunocompetent person, involving multiple bones and lungs, with *Cryptococcus neoformans* identified as the causative agent.

**Case presentation:**

An Indonesian man, previously in good health, presented with a chief complaint of successive multiple bone pain lasting for more one month, without any prior history of trauma. Additionally, he reported a recent onset of fever. On physical examination, tenderness was observed in the left lateral chest wall and right iliac crest. Laboratory findings indicated mildly elevated inflammatory markers. A computed tomography (CT) scan of the chest revealed an ovoid solid nodule in the right lower lung and multifocal osteolytic lesions in the sternum, ribs, and humeral head. A magnetic resonance imaging (MRI) study of the sacrum showed multiple lesions in the bilateral iliac bone and the lower L4 vertebral body. Confirmation of Cryptococcal osteomyelitis involved a fine-needle biopsy and culture, identifying *Cryptococcus neoformans* in the aspirate. The patient responded positively to targeted antifungal treatments, leading to a gradual improvement in his condition.

**Conclusions:**

This case emphasizes the need to consider *Cryptococcus neoformans* osteomyelitis in immunocompetent patients with bone pain. A definitive diagnosis involves a fine-needle biopsy for pathology and culture, and prompt initiation of appropriate antifungal treatment has proven effective in preventing mortality.

## Introduction

Cryptococcosis stands as a noteworthy global opportunistic infection, predominantly impacting immunocompromised individuals, including patients with human immunodeficiency virus (HIV), organ transplant recipients, and those with malignancies [1–3]. However, there are also documented instances of cryptococcal infections occurring in immunocompetent hosts [4, 5]. Cryptococcosis primarily affects the respiratory and central nervous systems (CNS) [2], with bone involvement being rare. Over 95% of cryptococcal infections are caused by *Cryptococcus neoformans*, while *Cryptococcus gattii* is responsible for the rest, particularly in immunocompetent hosts [1, 6]. Both thrive in environments with bird droppings, like contaminated soil [7]. Cryptococcus usually gain entry through the respiratory system, causing respiratory complications, and demonstrates a neurotropic inclination by selectively affecting the central nervous system [2]. Bone involvement occurs in less than 10% of disseminated Cryptococcosis cases [1, 8]. This report outlines an uncommon instance of disseminated Cryptococcosis in an immunocompetent individual, affecting multiple bones and lung attributed to *Cryptococcus neoformans.*

## Case presentation

A previously healthy 28-year-old married Indonesian male, employed in the textile industry in Taiwan, presented with one month of progressive bone pain. Initially, he reported posterior neck pain a month ago. About a week before admission, he experienced left-sided chest pain. He sought outpatient care, where Ibuprofen and dexamethasone tablets were administered for three days. Subsequently, he developed discomfort in the right iliac crest region, radiating down his back thigh to the calf. He was later hospitalized due to a one-day fever peaking at 40.6 °C, accompanied by chills. He did not report muscle weakness, numbness, or any urinary or fecal incontinence. Furthermore, he showed no night sweats, cough, or sputum production, and had not experienced trauma or weight loss. The patient had no history of antibiotic or immunosuppressant use, except for a three-day, total 3 mg course of dexamethasone prior to admission. He denied any recent exposure to soil or birds. He had not traveled in the past 3 years. Furthermore, he had no history of smoking or alcohol consumption. Additionally, there were no reported instances of intravenous drug use, blood product transfusions, or casual sexual activity in his medical history.

On physical examination, the patient presented as acutely ill, yet remained awake, alert, and oriented to time, place, and person. His vital signs were as follows: oral temperature 40.6 °C, pulse rate 147 beats/min, respiratory rate 21 breaths/min, and blood pressure 110/79 mmHg. Notably, respiratory, cardiac, and abdominal examinations yielded unremarkable findings. Nonetheless, tenderness was noted in the left lateral chest wall and right iliac crest. No redness or swelling was observed. The straight leg raising test was negative, and motor examination revealed normal strength (5/5 power) in both legs. No palpable lymph nodes or signs of oral candidiasis were observed.

**Fig. 1:**
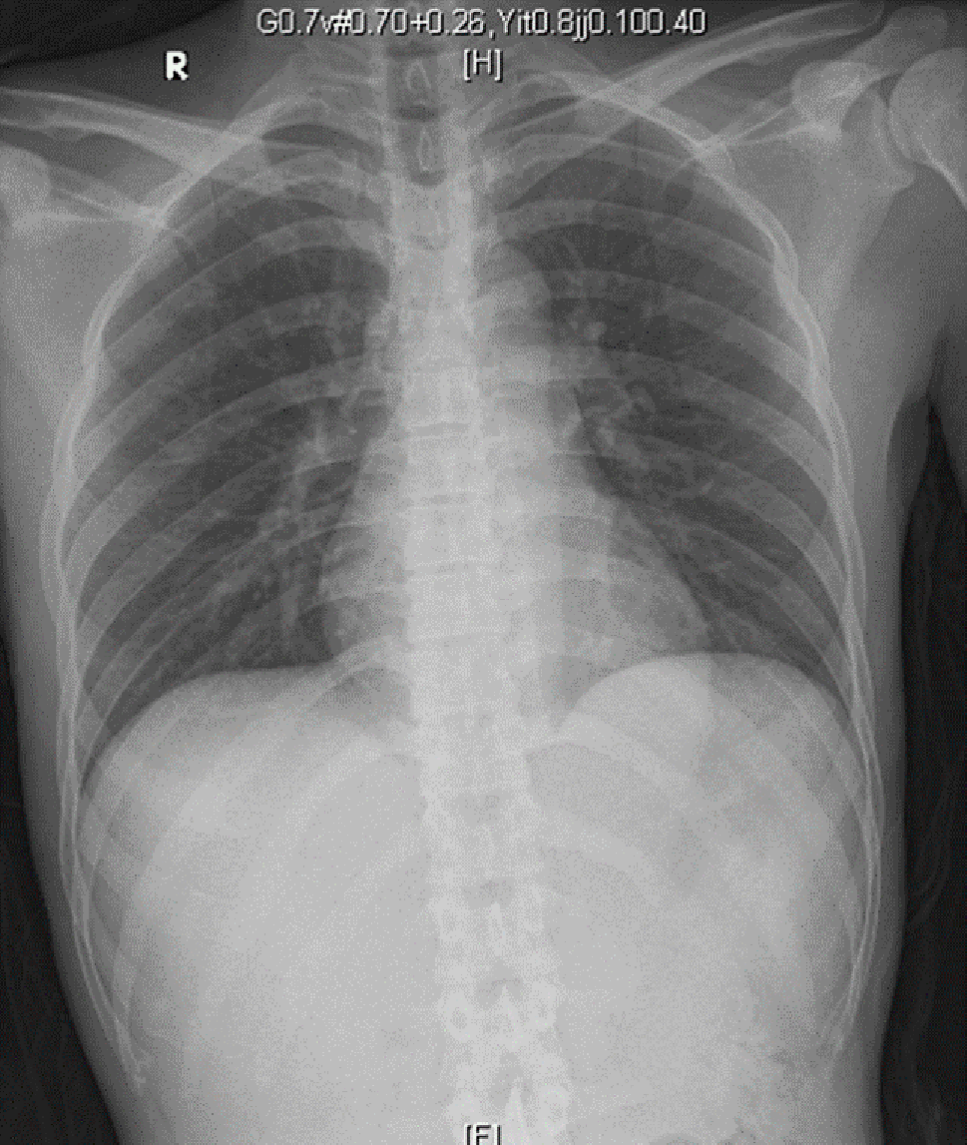
Chest X-ray showing mild increased lung markings in both lower lung fields

**Fig. 2:**
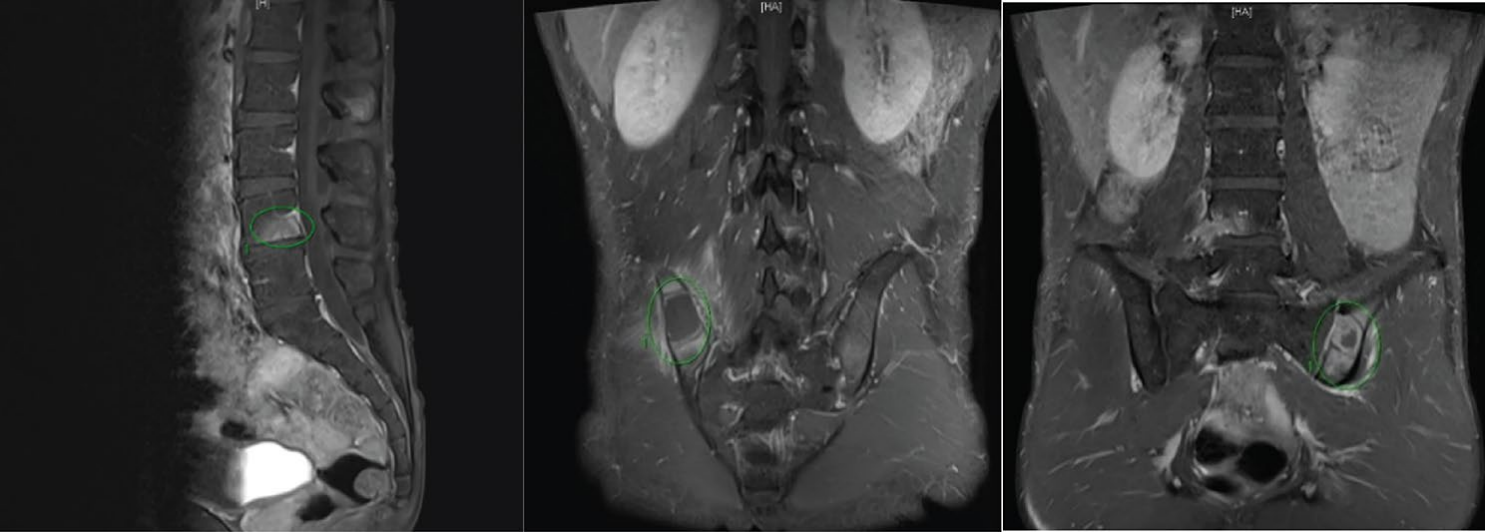
Magnetic resonance imaging (MRI) of the sacrum revealing diffuse decreased T1 signals affecting the thoracolumbar spine and bilateral iliac bones, along with multiple T2 hyperintensity lesions showing enhancement in the bilateral iliac bones and lower L4 vertebral body

**Fig. 3:**
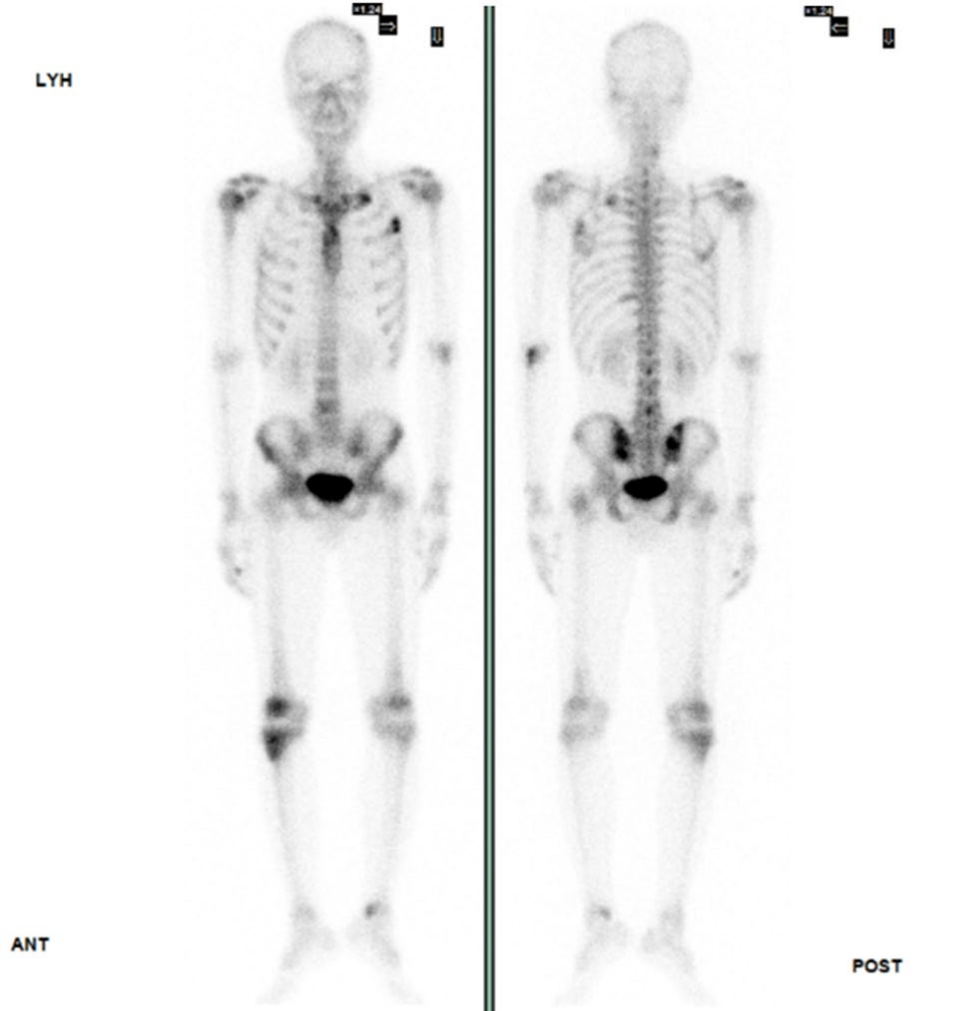
Tc-99 m whole-body bone scan revealing areas of increased activity in the left parietal bone, left lower cervical spine, L2, L4, bilateral sacroiliac joints, right knee, left ankle, and the anterolateral aspects of the left 1st, 3rd, and posterior left 10th ribs

**Fig. 4:**
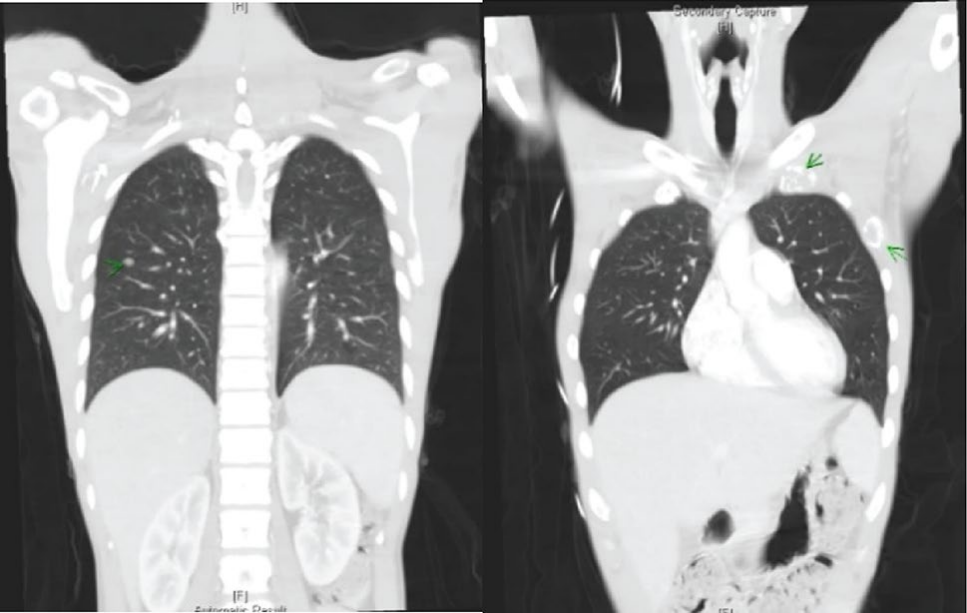
Chest computed tomography (CT) scan revealing a 0.85 cm ovoid solid nodule in the right lower lung, along with multifocal osteolytic bony lesions observed in the sternum, left 1st, 3rd ribs, and right humeral head

**Fig. 5:**
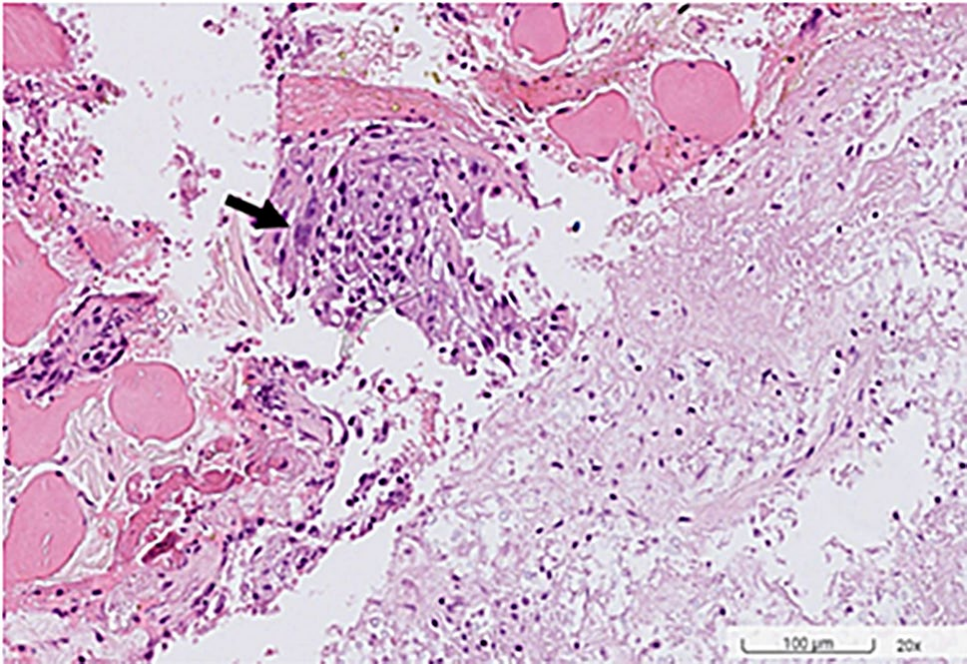
This section reveals necrosis with yeast cells and focal aggregate of epithelioid histiocytes with multinucleated giant cells (arrow), suggesting granulomatous inflammation

**Fig. 6:**
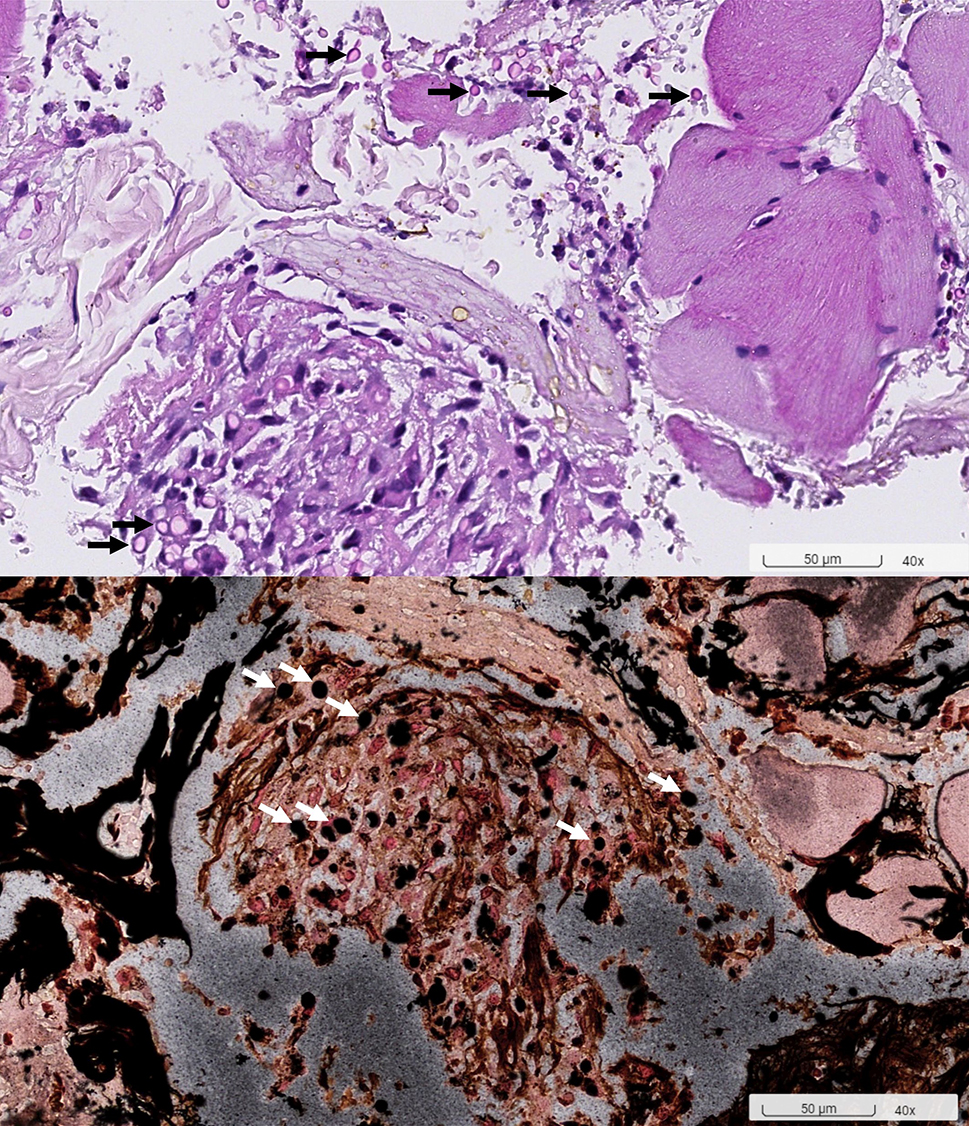
The special stains of PAS (upper) and GMS (lower) highlight the yeast cells of cryptococcus (arrows)

Table 1 displays laboratory findings revealing an elevated erythrocyte sedimentation rate (ESR) of 64 mm/h (reference range 0–15), a slightly increased C-reactive protein (CRP) level of 2.82 mg/dL (reference range < 1). SARS-CoV-2 RNA and Influenza antigen tests were negative. White blood cell count: 8.3^3/µL (reference range 3.5–9.1), 71.6% neutrophils (reference range 39.4–72.6%), 11.7% lymphocytes (reference range 21–51%). Hemoglobin: 12.5 g/dL (reference range 14–17). Elevated alanine aminotransferase (ALT): 66 U/L (reference range 11–42). Alkaline phosphatase (ALP): 71 U/L (reference range 34–104). Bilirubin: 0.4 mg/dL (reference range 0.3–1.0). Glucose: 104 mg/dL (reference range < 140). ANA reactivity: positive at ≧ 1:160 (reference range 1:80(-)). Complement C3: 163.4 mg/dL (reference range 87.0–200.0). Complement C4: 46.2 mg/dL (reference range 13.1–50.2). Tumor markers: CA199 8.5 U/mL (reference range ≦ 35.0), CEA 1.5 ng/mL (reference range < 5.0 non-smoker), PSA 1.117 ng/mL (reference range ≦ 4.0). Urinalysis and blood cultures: negative. Results of the serum biochemistry tests were essentially normal. Assessment for an underlying immunodeficiency was negative for HIV and autoantibodies against interferon-gamma (IFN-γ) or granulocyte-macrophage colony-stimulating factor (GM-CSF). Lumbar puncture (LP) showed clear, colorless cerebrospinal fluid (CSF) with an opening pressure of 65 mmH_2_O. CSF parameters were normal: cell count < 4/µL (reference range, 0–5), glucose 69 mg/dL (reference range, 40–70), and protein 44.3 mg/dL (reference range, 15–45). Cryptococcus polymerase chain reaction (PCR) was negative. India ink staining was not done. CSF and blood cultures were negative for bacterial or fungal growth. Chest X-ray showed increased lung markings in lower lung fields (Fig. 1). Lumbar spine X-ray ruled out compression fracture. Sacrum X-ray indicated mild narrowing of the L5-S1 disc. Liver echo indicated splenomegaly of unknown cause. Nerve conduction studies (NCS) of the lower limbs were normal. Magnetic resonance imaging (MRI) of the sacrum showed multiple lesions in the bilateral iliac bone and lower L4 vertebral body (Fig. 2). A 99mTc whole body bone scan indicated an increased uptake in the left parietal bone, left lower cervical spine, L2, L4, bilateral SI joint, right knee, left ankle, anterolateral left 1st and 3rd ribs, and posterior left 10th rib (Fig. 3). Furthermore, a chest computed tomography (CT) scan showed a 0.85 cm ovoid solid nodule in the right lower lung and multiple osteolytic bony lesions in the sternum, left 1st and 3rd ribs, and right humeral head (Fig. 4). A CT-guided biopsy confirmed Cryptococcus infection in the L4 vertebral body and right iliac bone. Fine-needle aspiration cytology, stained with Grocott’s-Gomori Methenamine silver (GMS), revealed Cryptococcus. Histopathological analysis confirmed a granuloma consistent with Cryptococcosis (Figs. 5 and 6), and cultures from the aspirate yielded *Cryptococcus neoformans* at both sites. The patient’s serum Cryptococcal antigen titer of 1:2560 confirmed disseminated Cryptococcosis, suggesting probable involvement in the sternum, left 1st and 3rd ribs, right humeral head, right lower lung, and definitively affecting the L4 spine and both iliac bones. After admission, the patient received IV amoxicillin/clavulanate (1500 mg every 8 h). Persistent fever and bone pain indicated Cryptococcosis, leading to subsequent treatment with IV amphotericin B (0.7 mg/kg/day) and oral flucytosine (100 mg/kg/day in 4 divided doses) for 4 weeks. Upon improvement, the patient was discharged with a one-year prescription for oral fluconazole (400 mg/day). Inflammatory markers, such as ESR and CRP, consistently remained within normal limits, and the patient remained symptom-free post-discharge.

**Table 1:** Laboratory results

Parameter	Value	Reference range
ESR	64 mm/h	0–15
CRP	2.82 mg/dL	< 1
SARS-CoV-2 RNA	negative	
Influenza	negative	
WBC count	8.3 × 10^3/µL	3.5–9.10.0
Neutrophil	71.6%	39.4–72.6
Lymphocyte	11.7%	21–51
Hemoglobin	12.5 g/dL	14–17
ALT	66 U/L	11–42
ALP	71 U/L	34–104
Bilirubin	0.4 mg/dL	0.3-1.0
Glucose	104 mg/dL	< 140
ANA activity	1: 160	1: 80 (-)
Complement C3	163.4 mg/dL	87.0-200.0
Complement C4	46.2 mg/dL	13.1–50.2
CA199	8.5 U/mL	< 35
CEA	1.5 ng/mL	< 5, non-smoker
PSA	1.17 ng/mL	<=4
Urine analysis	negative	
HIV Ab	negative	
Anti-IFN-γ Ab	negative	
Anti-GM-CSF Ab	negative	

## Discussion

Cryptococcosis, a potentially fatal fungal infection, primarily affects immunocompromised individuals worldwide, especially those with HIV or post-solid organ transplantation [1, 2]. Usually gaining entry through the lungs, Cryptococcus frequently results in pneumonia and meningitis [2]. Cryptococcal osteomyelitis is uncommon, typically stemming from a primary pulmonary infection that spreads through the bloodstream [8, 9] or, less commonly, through traumatic inoculation via the skin [10]. The patient experienced one month of widespread bone pain and a new onset of fever, with no evidence of respiratory infection or prior trauma. A CT scan of the chest found a radiopaque nodule in the right middle lung field, challenging to biopsy due to its sub-centimeter size. The spread of infection from the lungs to blood stream and disseminated to the bones is a possible explanation. The individual displayed no identified risk factors for immunocompromise, including but not limited to diabetes (random glucose: 104 mg/L), sarcoidosis, malignancy (CA199: 8.5U/mL; CEA: 1.5ng/mL; PSA: 1.117 ng/mL), solid organ transplant, or prolonged steroid usage. In Kuo et al.‘s investigation, six of 23 patients with disseminated Cryptococcus harbored anti-GM-CSF autoantibodies, and all five with positive culture reports were infected with *Cryptococcus gattii* [11]. The association between the presence of anti-interferon-γ autoantibodies and the onset of immunodeficiency with intracellular infections has been clearly established [12–15]. Cryptococcus, typically extracellular, evades the host immune system by forming phagosomes, and preventing phagocytosis through “titan cells” formation [16, 17]. Consequently, our assessment for immunodeficiency related to HIV and the presence of auto-antibodies against IFN-γ or GM-CSF yielded negative results. Despite ANA reactivity ( ≧ 1:160+), low-titer ANA can occur in subacute/chronic infection. Cryptococcal osteomyelitis, primarily caused by *Cryptococcus neoformans*, typically affects immunocompromised individuals [18, 19], including those with sarcoidosis, tuberculosis, steroid therapy, or diabetes mellitus [20]. However, it can also occur in immunocompetent individuals [10, 21–23]. Apparently, the patient has not disclosed any immune deficiency. Disseminated *Cryptococcus neoformans* infection involving multiple bones and lungs was diagnosed. It’s important to note that certain immune deficiencies may only become apparent through advanced investigations that are currently beyond our reach. Clinical presentation, bone pain, and osteolytic lesions resembled metastatic malignancy. Definitive diagnosis via fine-needle aspiration biopsy and culture revealed Cryptococcal osteomyelitis. Imaging lacks typical features, but previous reports document lesions mimicking malignancy [24].

## Conclusions

In immunocompetent hosts presenting with bone pain and osteolytic lesions, Cryptococcal osteomyelitis should be included in the differential diagnosis. The definite diagnosis should be confirmed through FNAC and fungal culture, with further investigation into immunological assessments recommended.

## Data Availability

All relevant data are within the paper and its supporting information files.

## Abbreviations

CT: computed tomography
MRI: Magnetic resonance imaging
HIV: human immunodeficiency virus
CNS: central nervous system
SLRT: Straight leg raising test
ESR: erythrocyte sedimentation rate
CRP: C-reactive protein
FNAC: fine-needle aspiration cytology
ANA: antinuclear antibody
CA199: carbohydrate antigen 19 − 9
CEA: carcinoembryonic antigen
PSA: Prostate-Specific Antigen
IFN-γ: Interferon gamma
GM-CSF: granulocyte-macrophage colony-stimulating factor
CSF: Cerebrospinal fluid
PCR: polymerase chain reaction
